# The relationship between neonatal stress in preterm infants and developmental outcomes at the corrected age of 24–30 months

**DOI:** 10.3389/fpsyg.2024.1415054

**Published:** 2024-05-22

**Authors:** Halyna Pavlyshyn, Iryna Sarapuk, Kateryna Kozak

**Affiliations:** Department of Pediatrics, I. Horbachevsky Ternopil National Medical University, Ternopil, Ukraine

**Keywords:** developmental status, preterm infants, neonatal NICU-related stress, cortisol, melatonin

## Abstract

**Aim:**

The aim of research was to study the relationship between the stress experienced by preterm infants in the neonatal intensive care unit (NICU) and developmental status in the follow up, and to establish factors, associated with their neurodevelopment.

**Methods:**

The first stage of research involved measuring stress markers (cortisol, melatonin) in infants (*n* = 56) during their NICU stay; the second phase assessed the developmental status at the corrected age of 24–30 months.

**Results:**

The total ASQ-3 score, communication, problem solving, and personal-social skills scores at the corrected age of 24–30 months were positively correlated with melatonin level determined in the neonatal period (*r* = 0.31, *p* = 0.026; *r* = 0.36, *p* = 0.009; *r* = 0.30, *p* = 0.033, and *r* = 0.32; *p* = 0.022 respectively). In the same time, ASQ-3 communication and personal-social scores were negatively correlated with cortisol level (*r* = −0.31, *p* = 0.043; *r* = −0.35, *p* = 0.022). The ROC-curve analysis revealed that a decrease of melatonin below 3.44 ng/mL and 3.71 ng/mL during the neonatal period could predict communication and problem-solving delay, respectively. An increase in cortisol above 0.64 mcg/dl is predictive in personal-social delay. Negative correlation was identified between the NICU and total hospital stay duration and ASQ-3 communication scores in the follow-up (*r* = −0.27; *p* = 0.049 and *r* = −0.41; *p* = 0.002, respectively). The duration of mechanical ventilation was negatively correlated with gross motor scores (*r* = −0.46; *p* = 0.043). Apgar score was positively correlated with ASQ-3 communication (*r* = 0.29; *p* = 0.032) and personal-social scores (*r* = 0.28; *p* = 0.034); maternal age—with ASQ-3 total (*r* = 0.29; *p* = 0.034), communication (*r* = 0.37; *p* = 0.006), and personal-social scores (*r* = 0.29; *p* = 0.041). Positive correlations were observed between gestational age and communication scores (*r* = 0.28; *p* = 0.033). Infants who suffered neonatal sepsis had significantly often delay of communication (*p* = 0.014) and gross motor skills (*p* = 0.016). Children who required mechanical ventilation were more likely to have communication delay (*p* = 0.034).

**Conclusion:**

Developmental outcomes in preterm infants at the corrected age of 24–30 months were associated with neonatal stress. Correlations between the communication, problem-solving and personal-social development in the follow up and cortisol and melatonin levels determined in the neonatal period supported this evidence. Factors as low gestational age, duration of hospital and NICU stay, mechanical ventilation, and sepsis were associated with more frequent delays in communication, gross motor and problems-solving skills.

## Introduction

Survival of extremely preterm infants has increased significantly over the past two decades (Younge et al., 2017) and is 74% in Norway (Stensvold et al., 2017) and 78.3% in the USA (Bell et al., 2022). In Ukraine, the survival of newborns with extremely and very low birth weight in the early neonatal period was 70.2% in 2020 (Znamenska et al., 2022). Despite the increase in the survival rate of very and extremely preterm newborns, the frequency of neonatal morbidity with long-term outcomes and complications associated with premature birth does not decrease (National Guideline Alliance (UK), 2017). The NICE guideline summarizes that infants born prematurely can be at risk of developing a range of motor problems, including cerebral palsy, executive functioning, learning, speech, language, sensorineural hearing loss, blindness, mental retardation, autism spectrum disorders, feeding and eating difficulties (National Guideline Alliance (UK), 2017; Chung et al., 2020; Morgan et al., 2022). Regular follow-up assessment allowed identifying more and more disorders of neuropsychological and social–emotional development in this cohort of children. These disorders include learning disabilities, low average IQ scores, attention deficit hyperactivity disorder, neuropsychological deficits, impairments in visual-motor integration and executive functions, various temperamental difficulties, emotional problems, and regulatory disorders (Hollanders et al., 2019; Jois, 2019). These problems are related to gestational birth age as well as neonatal, biological and maternal factors. Among them are: gender, birth weight, neonatal sepsis, retinopathy of prematurity, bronchopulmonary dysplasia, neonatal surgery, antenatal and postnatal steroids, access to breastmilk in the neonatal/infant period, socioeconomic status, and exposure to neonatal stress and pain (Synnes et al., 2017; Cheong and Preterm Follow Up Guideline Development Group, 2023).

Problem of neonatal pain, discomfort, and stress remains relevant in neonatal intensive care units (NICUs), especially for preterm infants (Kothari et al., 2016). Their first days and sometimes months of life are full of painful and harmful procedures in an overstimulating environment where they are physically separated from their mothers. It is quite difficult to differentiate between stress and pain in the neonatal period (Abdallah and Geha, 2017). Every painful event is believed to be stressful, but not all stress is painful. At the same time stress is an important factor influencing how infants perceive and respond to pain (Jones et al., 2018). Some authors concluded that early and frequent pain perception in the youngest infants was associated with the development of a persistent stress state (Grunau, 2013).

Neonates experience numerous painful manipulations in the intensive care unit, undergoing 10 to 18 painful procedures per day (Cruz et al., 2016; Victoria and Murphy, 2016). At the same time, in 42–100% of cases, infants are exposed to painful manipulations without any form of anesthesia (Cruz et al., 2016). The neurobiological vulnerability to pain in preterm infants is well established due to their lower pain threshold, sensitization to repeated pain, and immature homeostatic systems (Grunau et al., 2009). Uncontrolled and long-lasting pain and pain-related stress can lead to a sensitization phenomenon in extremely and very preterm infants, resulting in persistent dysregulation of stress response systems (Provenzi et al., 2015), and significant short-term and long-term adverse outcomes. The early consequences of painful procedures cause the disturbances of vital parameters (heart rate variability, desaturation, apnea, arterial hypertension, intracranial pressure fluctuations) (Lavanga et al., 2021), which together deplete insufficient energy reserves of a preterm child, increasing the risk of morbidity and mortality (Hatfield, 2014; Walker, 2019).

The experience of pain and stress in the neonatal period forms a somatosensory basis for further perceptual, cognitive and social development. Excessive and prolonged exposure to stress during NICU stay may exceed the infant’s natural regulatory capacity, and may permanently alter neuroendocrine, autonomic, cardiovascular, and neuronal functions (Lammertink et al., 2021). The mismatch between the brain development needs of preterm infants and the realities of NICU period treatment causes severe neurophysiological, psycho-emotional, and psychosocial developmental problems, resulting in persistent neurological and psychiatric morbidities throughout life. A range of long-term consequences such as visual impairment/blindness, hearing impairment/deafness, cerebral palsy, developmental delay and intellectual impairment in childhood and adulthood are attributed to painful medical procedures received early in life, during a critical period of neurological development (Valeri et al., 2015; Burnett et al., 2018; Chau et al., 2019). In general, the NICU stay of a preterm infant is a potentially toxic stressor with negative outcomes for brain architecture and neuroendocrine function throughout the life, even in the absence of serious clinical complications (Montirosso et al., 2016).

A long-term response to chronic stress leads to prolonged activation of the hypothalamic-pituitary-adrenal and sympathetic nervous systems, increasing levels of glucocorticoids and catecholamines (Liang and Booker, 2024). Glucocorticoids bind to receptors in the hippocampus, are able to change its structure and function and, at elevated levels, impair neurogenesis, increase vulnerability to strokes, and reduce dendritic branching (Chetty et al., 2014; Finegood et al., 2017). Corticosteroids might decrease the activity of N-acetyl-transferase during stress events, and hence, raised levels of cortisol may negatively influence synthesis of melatonin (Sertaridou et al., 2018). This neuro-hormone protects the developing brain of newborn infants being able to prevent abnormal myelination and inflammatory glial reaction, which are the main causes of white matter damage (Garofoli et al., 2021). The neuroprotective effect of melatonin also manifests due to its antioxidant properties. Melatonin provides not only a direct neuro-protective effect, but also protects the body from excessive stress, especially pain-induced stress with all its negative long-term consequences, showing analgesic properties (Perrone et al., 2023). It is shown that peak melatonin levels occur when cortisol levels are at their lowest (Sertaridou et al., 2018).

### The aim of this research

The aim of this research was to study the relationship between the stress experienced by the preterm infants in the NICU and developmental status at the corrected age of 24–30 months. The secondary objective was to establish factors that were associated with developmental delay of those patients.

## Methods

### Study design

The study design included two stages. At the first stage, the stress markers (cortisol, melatonin) were investigated in preterm infants during their NICU (level III NICU of Ternopil Regional Perinatal Center, Ukraine) stay at the early neonatal period (the first week of life).

At the next stage, development assessment was carried out at the corrected age of 24–30 weeks in the follow-up center of Ternopil Regional Children’s hospital by the ASQ-3 questionnaire.

### Recruitment and randomization

There were 84 eligible infants during the first study period, with 72 recruited (2 infants did not meet the inclusion criteria, 4 neonates were excluded because of insufficient investigated sample (saliva), and 6—parents declined to participate). Prematurity (GA ≤ 34 weeks) was the criteria for inclusion in this study. Exclusion criteria were the following: chromosomal disorders, congenital malformations.

At the second stage of the study of 72 recruited infants at the neonatal period, developmental assessment was carried out for 58 children according to the follow-up program. Two extremely preterm infants of recruited 72 children died in the late neonatal period, 9 children did not visit the follow up examination due to transfer to other countries (because of the war in Ukraine), 3 mothers refused to pass developmental status screening tests. Two of 58 examined children at the corrected age 24–30 months were diagnosed with cerebral palsy, and thus were excluded from the study.

Thus, infants who withdrew from the study (*n* = 16), or did not survive to the corrected age 24–30 months (*n* = 2), or whose parents refused to participate at the first or second stage of the research (*n* = 10) were not included in the analysis as no outcomes could be obtained. The characteristics of the 56 infants included in the study are presented in the results chapter of the manuscript.

### Sample collection and stress markers assay

We examined the level of salivary cortisol as the main stress marker and the level of urinary melatonin that had anti-stress properties. Saliva samples were collected by using the cotton sponges, after that were extracted from the sponges by centrifugation (2 min at 2000 × g). Saliva samples were collected without the usage of any salivation stimulating agents. After extraction samples were frozen and stored at −20°C. Enzyme Immunoassay for the quantitative determination of free cortisol in human saliva was used to analyze cortisol levels in the samples (IBL International GmbH, Hamburg, Germany).

Urine was collected by using the cotton sponges and after that was extracted from the sponges by centrifugation (2 min at 2000 × g). After extraction, urine samples were centrifuged for 20 min at 1000 × g at 2–8°C and after that were frozen and stored at-80° C. Enzyme Immunoassay for the quantitative determination of melatonin sulfate was used to analyze melatonin levels in the urine samples (Elabscience, Wuhan, China). Samples were analyzed in duplicate, and assays were performed using provided controls according to the manufacturer’s instructions.

Developmental status assessment. Neurodevelopmental outcomes of children at the corrected age of 24–30 months were assessed by using the ASQ-3 questionnaires (Ages and Stages Questionnaire-3rd Edition). The ASQ is a screening tool that assesses a child’s developmental status in five areas of development: communication, gross motor, fine motor, problem solving, and personal-social development, helping to identify impairments in the early stages. Each questionnaire contains 30 questions addressing five specific developmental domains and one general section that focuses on common concerns for parents. Parents completed the questionnaire after they obtained detailed instructions on how to fill in it. Parents observed their child’s skills and answer “Yes” (score: 10), “Sometimes” (score: 5) and “Not yet” (score: zero) to six questions in each of the five domains. At the next stage, the summed scores achieved in each developmental area were compared with threshold values for different age groups. Both the sum of points for each area of development and the frequency of delays in each area were evaluated. The total score for each area of development is the sum of all items in the area, ranging from zero to 60, with higher scores indicating better development (Squires et al., 2009). Cronbach’s alpha coefficients for the Ukrainian version of the ASQ-3 indicated it good consistency (0.847).

### Ethics statement

The local ethical committee of I. Horbachevsky Ternopil National Medical University approved the study. All the participants who took part in the study signed the informed consent.

### Statistics

All computations were performed using StatSoft STATISTICA Version 13 (Tulsa, OK) and IBM SPSS Statistics 21. Quantitative data were presented as the median and interquartile range (IQR; 25th to 75th percentiles). For qualitative parameters, absolute and relative frequencies were presented. Age, anthropometric measures, and scores of ASQ-3 questionnaire results were presented as mean (Mean) and standard deviation (SD). The Mann–Whitney U-test (for two independent groups) was used to compare numerical data. Proportions were compared between the two groups using the two-tailed Fisher exact test. Significance was assumed at *p* < 0.05. To assess the influence of the factor on the result, the Odds Ratio, its 95% confidence interval, and the confidence level were calculated. Spearman correlations were used to assess the associations among measures. ROC-curves were analyzed to define biochemical predictors of neurodevelopmental outcomes. Sensitivity and specificity for diagnostic tests were evaluated.

## Results

Fifty-six preterm infants with a gestational age of less than 34 weeks participated in the study. There were 26 (46.4%) males and 30 (53.6%) females. The mean gestational age was (30.91 ± 2.47) 31.0 [29.0; 33.0] weeks. There were 10 (17.9%) extremely, 30 (53.6%) very preterm, and 16 (28.5%) moderate preterm infants. The mean birth weight was (1539.4 ± 443.7) g. There were 8 (14.3%) extremely low birth weight infants, 20 (35.7%)—very low birth weight, and 28 (50.0%)—low birth weight. Five newborns were small for gestational age. The main characteristics of infants (pregnancy and delivery history, early neonatal period) are shown in Table 1.

**Table 1:** Characteristics of preterm infants in the neonatal period.

Parameter	Statistical indicators	Study group
		*n* = 56
Maternal factors during pregnancy		
Maternal age	Mean ± SD	29.3 ± 4.4
Gravida		
1	[*n* (%)]	27 (48.2%)
≥2	[*n* (%)]	29 (51.8%)
Preeclampsia, eclampsia, gestational hypertension	[*n* (%)]	17 (30.4%)
Thyroid gland disorders	[*n* (%)]	6 (10.7%)
Acute respiratory viral infection	[*n* (%)]	9 (16.1%)
Polyhydramnios	[*n* (%)]	10 (17.9%)
Urinary tract infections	[*n* (%)]	14 (25.0%)
Multiple pregnancies	[*n* (%)]	17 (39.4%)
Maternal bleeding	[*n* (%)]	7 (12.5%)
Mode of delivery		
C-section	[*n* (%)]	44 (78.6%)
Characteristics of infants in the early neonatal period neonatal period		
Apgar score at the 1st min	Me [Lq; Uq]	6.0 [6.0; 7.0]
Apgar score at the 5th min	Me [Lq; Uq]	7.0 [7.0; 7.0]
Apgar score at the 1st min < 7 points	[*n* (%)]	29 (51.8%)
Apgar score at the 5th min < 7 points	[*n* (%)]	9 (16.1%)
Primary resuscitation	[*n* (%)]	31 (55.4%)
Surfactant replacement therapy	[*n* (%)]	20 (35.7%)
Respiratory distress syndrome (RDS)	[*n* (%)]	40 (71.4%)
Early-onset infection	[*n* (%)]	13 (23.2%)
Intraventricular hemorrhage	[*n* (%)]	19 (26.8%)
Mechanical ventilation	[*n* (%)]	20 (35.7%)
Continuous positive airway pressure	[*n* (%)]	46 (82.1%)
Late-onset sepsis	[*n* (%)]	14 (25.0%)
Necrotizing enterocolitis	[*n* (%)]	7 (12.5%)
Broncho-pulmonary dysplasia (BPD)	[*n* (%)]	7 (12.5%)
Retinopathy of prematurity ≥ III grade	[*n* (%)]	5 (8.9%)
Neonatal seizures	[*n* (%)]	13 (23.2%)
Duration of NICU treatment, days	Me [Lq; Uq]	9.0 [6.0; 15.0]
Duration of total hospital stay, days	Me [Lq; Uq]	31.5 [25.0; 41.0]
Duration of mechanical ventilation, days	Me [Lq; Uq]	8.5 [5.5; 19.5]

Assessment of the preterm infants’ stress in the NICU was carried out by measuring the level of the stress hormone cortisol and the anti-stress hormone melatonin. The level of cortisol in infants was 0.427 [0.251; 1.236] μg/dL. The level of melatonin in infants was 4.42 [2.15; 6.64] ng/mL. It was established that these markers were reliably negatively correlated with each other (*r* = −0.40; *p* = 0.013).

Developmental status assessment of preterm infants at the corrected age of 24–30 months showed that 18 infants (32.1%) had delayed communication development, 4 (7.1%)—delay in the gross motor development, 10 (17, 9%)—delay in the fine motor skills, 15 (26.8%)—delayed problem solving skills, 7 (12.5%)—delayed personal-social development. Fifteen (26.8%) children had delay in one area of development, 9 (16.1%)—in 2 areas, and 6 (10.7%) infants—in 3 or more areas. Results of developmental status assessment of the studied cohort of children according to the ASQ-3 questionnaire scores are presented in the Table 2.

**Table 2:** Indicators of the developmental status of preterm infants at the corrected age of 24–30 months according to the results of the ASQ-3 questionnaire.

Developmental area	Mean ± SD
Communication score	43.84 ± 18.49
Gross motor score	56.34 ± 6.14
Fine motor score	51.25 ± 8.75
Problem solving score	43.12 ± 12.23
Personal-social score	48.48 ± 10.44
Total ASQ-3 score	243.03 ± 37.69

**Figure 1:**
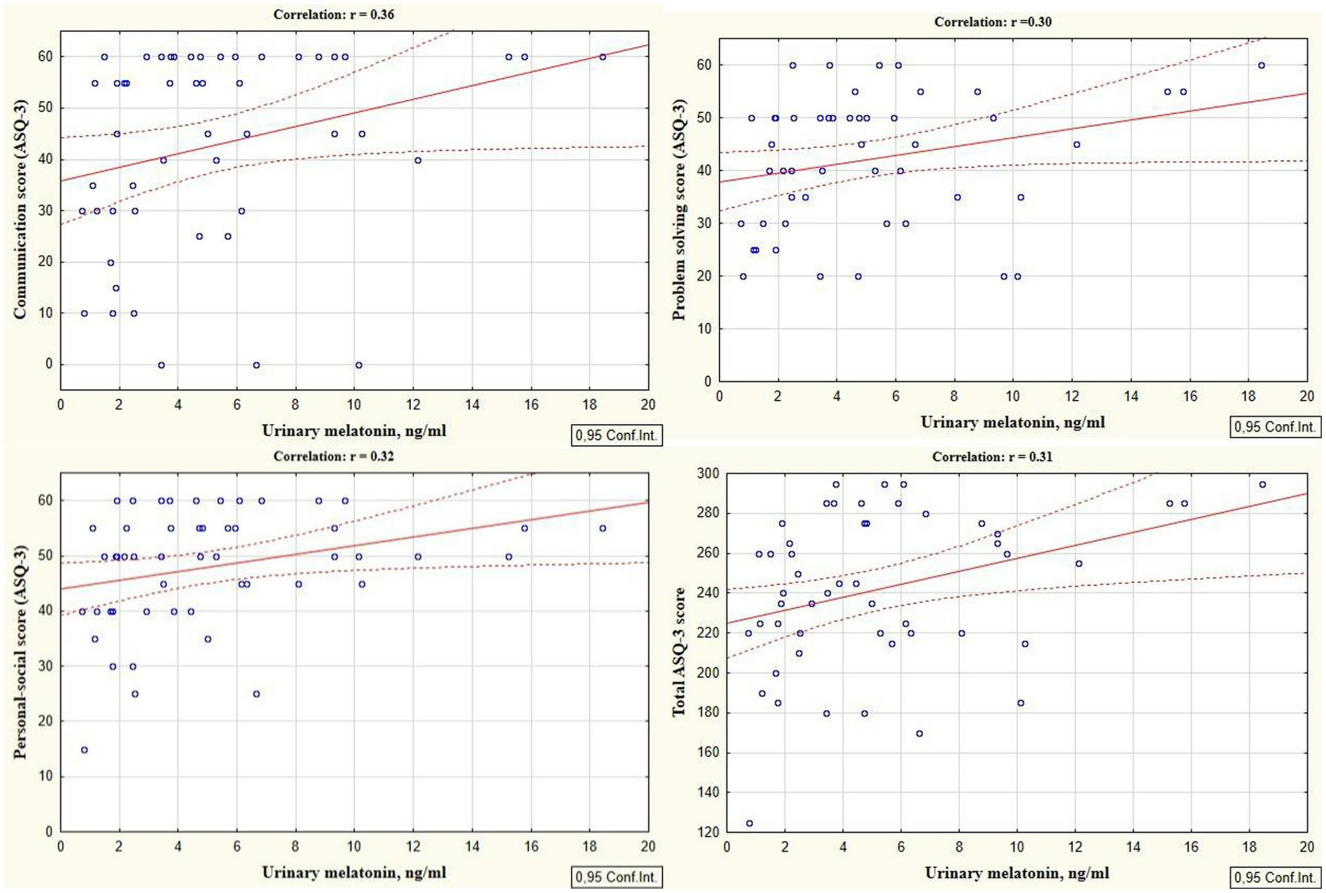
Correlations between ASQ-3 communication, problem solving, personal-social, and total scores of preterm infants at the corrected age of 24–30 months and melatonin level determined in the neonatal period.

**Figure 2:**
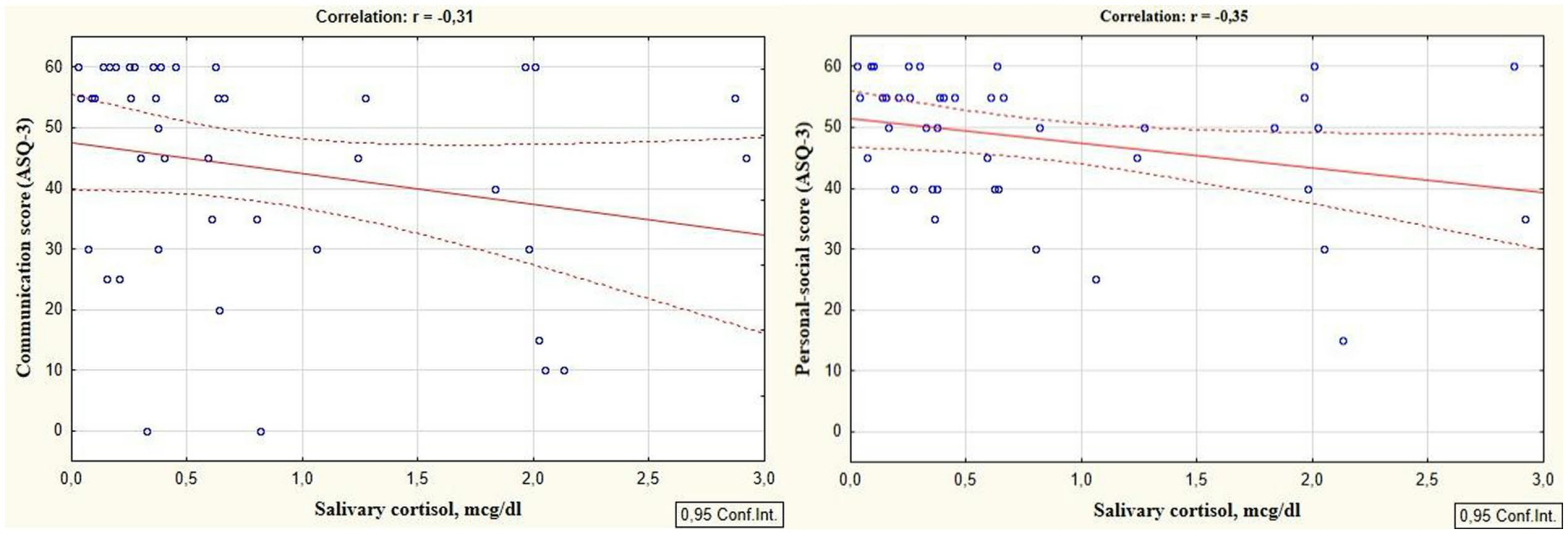
Correlations between ASQ-3 communication and personal-social scores of preterm infants at the corrected age of 24–30 months and cortisol level determined in the neonatal period.

Reliable correlations between the developmental areas scores of preterm infants at the corrected age of 24–30 months and levels of cortisol and melatonin determined in the neonatal period showed the relationship between the stress experienced by a preterm newborn in the NICU and the developmental status in the follow-up period. It was revealed that the total ASQ-3 score, communication, problem solving, and personal-social skills scores at the corrected age of 24–30 months were positively correlated with melatonin level determined in the neonatal period (*r* = 0.31, *p* = 0.026; *r* = 0.36, *p* = 0.009; *r* = 0.30, *p* = 0.033, and *r* = 0.32; *p* = 0.022 respectively), (Figure 1). At the same time, ASQ-3 communication and personal-social scores were negatively correlated with stress hormone cortisol (*r* = −0.31, *p* = 0.043; *r* = −0.35, *p* = 0.022, Figure 2).

**Figure 3:**
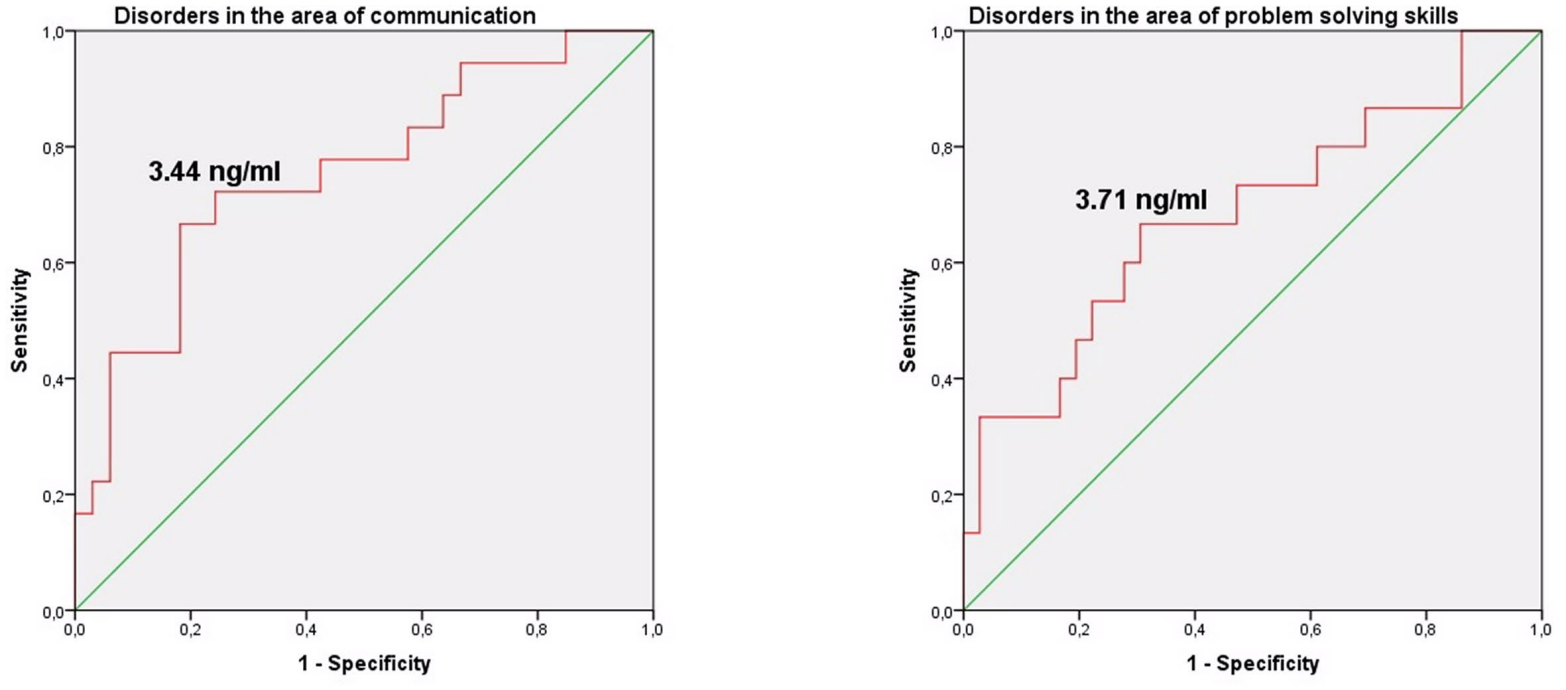
Melatonin level in the prediction of disorders in the area of communication and problem solving skills in preterm infants at the corrected age of 24–30 months.

**Figure 4:**
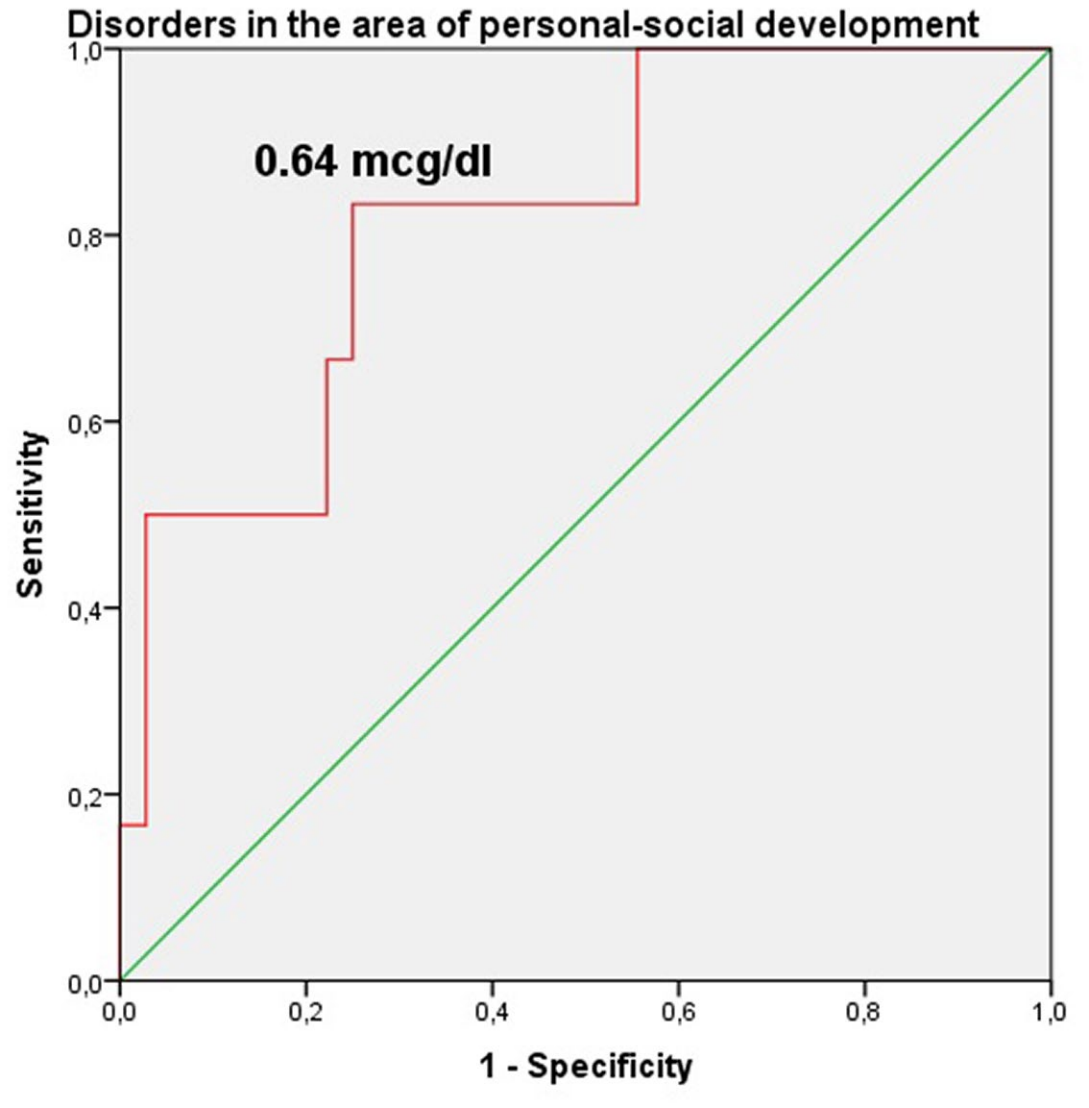
Cortisol level in the prediction of disorders in the area of personal-social development in preterm infants at the corrected age of 24–30 months.

The study identified cut-off points for developmental delays in preterm infants at the corrected age of 24–30 months based on neonatal levels of melatonin and cortisol. The ROC-curve analysis confirmed that decrease of the melatonin level below 3.44 ng/mL with sensitivity 0.72 and specificity 0.76 can predict the delay in communication development (AUC = 0.76). Melatonin level below 3.71 ng/mL can predict problem-solving skills delay (AUC = 0.68; sensitivity 0.67 and specificity 0.64, Table 3 and Figure 3). Increase cortisol above 0.64 mcg/dL in the neonatal period can predict the delay in personal-social skills (AUC = 0.82; sensitivity 0.83 and specificity 0.69, Table 4 and Figure 4).

**Table 3:** Melatonin level and developmental status outcomes in preterm infants at the corrected age of 24–30 months.

Groups	Communication delay	Gross motor skills delay	Fine motor skills delay	Problem solving skills delay	Personal-social development delay
AUC	0.76	0.72	0.52	0.68	0.71
95% CI AUC	0.61–0.99	0.46–0.97	0.33–0.72	0.51–0.85	0.50–0.91
p	0.003*	0.215	0.834	0.041*	0.082
Cut-off point	3.44	2.71	3.41	3.71	3.44
Sensitivity	0.72	0.67	0.44	0.67	0.71
Specificity	0.76	0.67	0.62	0.64	0.64

*statistically significant result.

**Table 4:** Cortisol level and developmental status disorders in preterm infants at the corrected age of 24–30 months.

Groups	Communication delay	Gross motor skills delay	Fine motor skills delay	Problem solving skills delay	Personal-social development delay
AUC	0.62	0.36	0.56	0.56	0.82
95% CI AUC	0.44–0.80	0.01–0.84	0.34–0.78	0.39–0.74	0.65–0.99
*p*	0.215	0.420	0.597	0.531	0.013*
Cut-off point	0.52	2.04	1.55	0.63	0.64
Sensitivity	0.64	0.33	0.38	0.46	0.83
Specificity	0.61	0.92	0.82	0.62	0.69

*statistically significant result.

**Figure 5:**
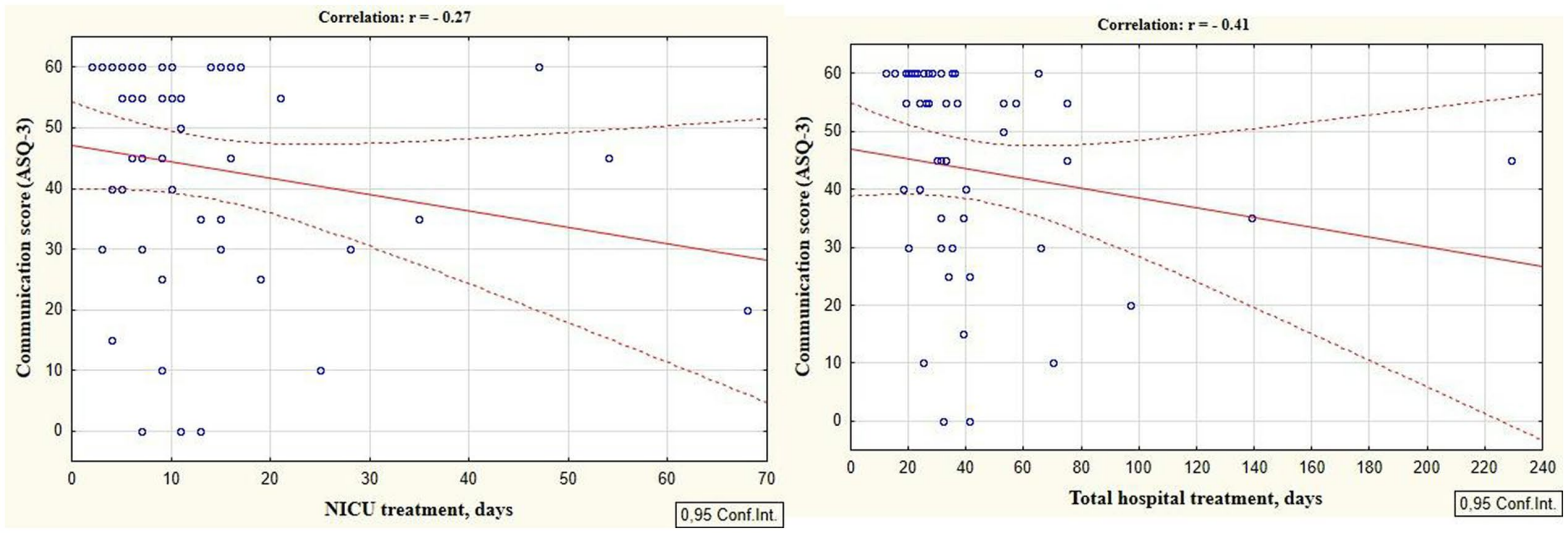
Correlations between ASQ-3 communication scores of preterm infants at the corrected age of 24–30 months and the duration of hospital treatment in the neonatal period (NICU and total hospital stay).

**Figure 6:**
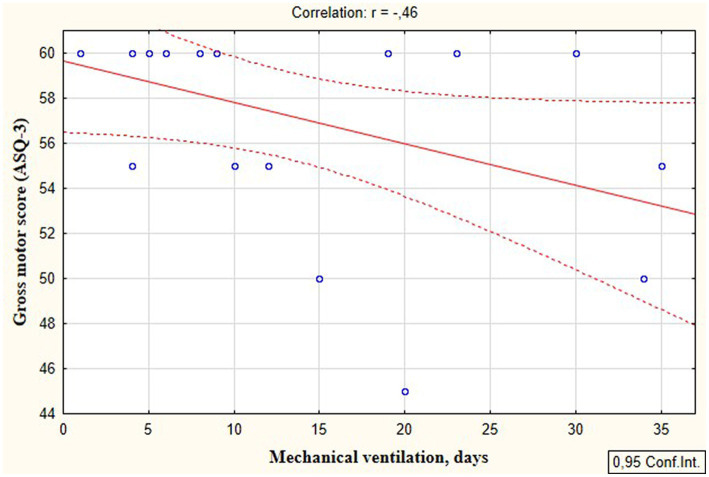
Correlations between ASQ-3 gross motor development scores of preterm infants at the corrected age of 24–30 months and the duration of mechanical ventilation in the neonatal period.

**Figure 7:**
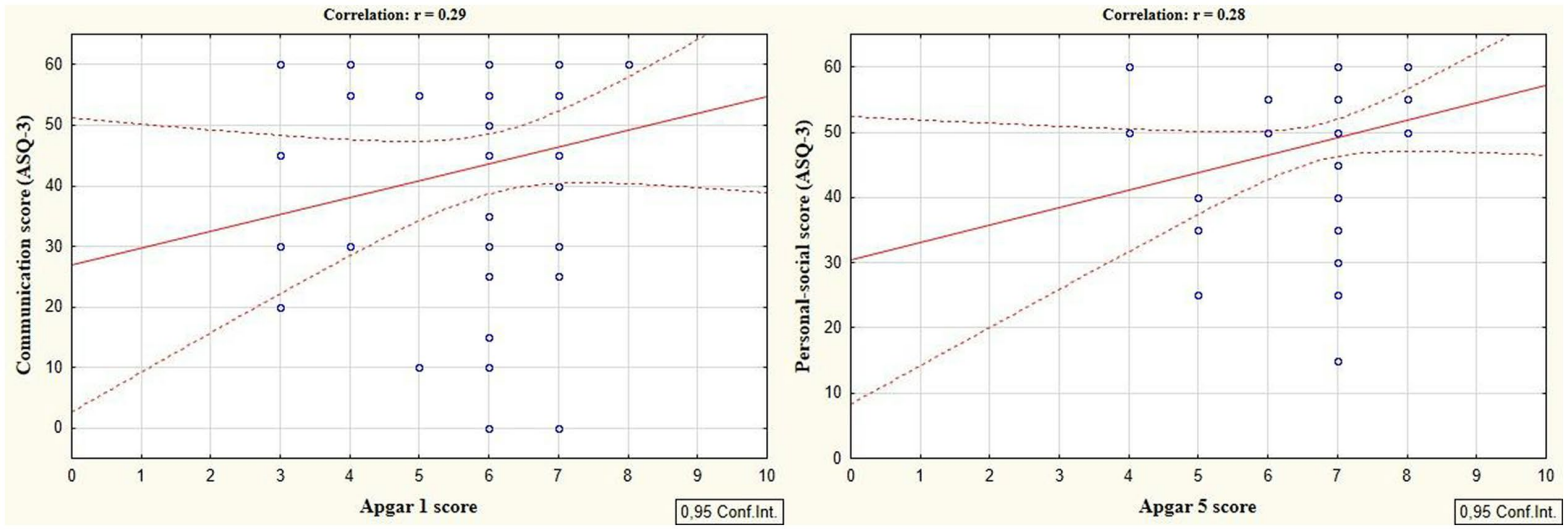
Correlations between ASQ-3 communication and personal-social scores of preterm infants at the corrected age of 24–30 months and the Apgar scores.

According to the secondary objective of our research, we studied factors that could be associated with developmental delay of preterm infants. Negative correlation was revealed between the duration of the NICU treatment, total duration of hospital stay and ASQ-3 communication scores at the corrected age of 24–30 months (*r* = −0.27; *p* = 0.049 and *r* = −0.41; *p* = 0.002, respectively, Figure 5). The duration of mechanical ventilation was negatively correlated with ASQ-3 gross motor scores (*r* = −0.46; *p* = 0.043, Figure 6). Apgar scores were positively correlated with ASQ-3 communication (*r* = 0.29; *p* = 0.032) and personal-social development scores (*r* = 0.28; *p* = 0.034, Figure 7). It was found the positive correlation between maternal age and ASQ-3 total (*r* = 0.29; *p* = 0.034), communication (*r* = 0.37; *p* = 0.006), and personal-social development scores of the child (*r* = 0.29; *p* = 0.041).

ASQ-3 communication scores of preterm infants were positively correlated with their gestational age (*r* = 0.28; *p* = 0.033). Extremely and very preterm infants significantly more often had delay in the area of communication (66.7% vs. 14.3%, *p* = 0.043; OR = 4.67; 95% CI 0.93–23.37; *p* = 0.061) and problem-solving development (60.0% vs. 0%, *p* = 0.003; OR = 20.06; 95% CI 1.12–358.58; *p* = 0.041) compared to moderately preterm infants.

It was shown that infants who suffered sepsis in the neonatal period had significantly more often delay in the communication development (52.4% vs. 20.0%, *p* = 0.014; OR = 4.40; 95% CI 1.34–14.38; *p* = 0.015), gross motor skills (23.5% vs. 0%, *p* = 0.016; OR = 18.25; 95% CI 0.93–358.49; *p* = 0.056) compared to those who did not have neonatal sepsis. The infants who were diagnosed with RDS in early neonatal period had significantly more often delayed fine motor skills (25.0% vs. 0%, *p* = 0.024; OR = 11.36; 95% CI 0.63–206.39; *p* = 0.101) compared to those who did not have RDS. Children who required mechanical ventilation in the neonatal period were significantly more likely to have delayed communication skills at the corrected age of 24–30 months (37.5% vs. 0%, *p* = 0.034; OR = 3.50; 95% CI 1.08–11.36; *p* = 0.037). The frequency of delay in each area of development in preterm infants depending on neonatal factors and morbidity are presented in Table 5.

**Table 5:** Developmental status outcomes in preterm infants at the corrected age of 24–30 months depending on neonatal factors and neonatal morbidities.

Neonatal factors and neonatal morbidities	Communication delay	Gross motor skills delay	Fine motor skills delay	Problem solving skills delay	Personal-social development delay
	*n* (%)	*n* (%)	*n* (%)	*n* (%)	*n* (%)
Gestation age					
<32 weeks (*n* = 40)	16 (40.0%)	3 (7.5%)	8 (20.0%)	15 (37.5%)	7 (17.5%)
33–34 weeks (*n* = 16)	2 (12.5%)	1 (6.25%)	2 (12.5%)	0	0
*р*	0.043*	0.679	0.406	0.003*	0.08
Gender					
Male (*n* = 26)	8 (30.8%)	2 (37.8%)	4 (15.4%)	8 (30.8%)	5 (19.2%)
Female (*n* = 30)	10 (33.3%)	2 (6.7%)	6 (20.0%)	7 (23.3%)	2 (6.7%)
*p*	0.533	0.638	0.463	0.372	0.156
Singleton (*n* = 39)	13 (33.3%)	3 (7.8%)	5 (12.8%)	11 (28.2%)	5 (12.8%)
Twins (*n* = 17)	5 (29.4%)	1 (5.9%)	5 (29.4%)	4 (23.5%)	2 (11.8%)
*p*	0.515	0.647	0.134	0.494	0.643
Mode of delivery					
Vaginal (*n* = 12)	5 (41.7%)	2 (16.7%)	2 (16.7%)	3 (25.0%)	1 (8.3%)
Cesarean section (*n* = 44)	13 (29.5%)	2 (4.5%)	8 (18.2%)	12 (27.3%)	6 (13.6%)
*p*	0.321	0.198	0.637	0.595	0.531
Apgar score 5 min					
<7 points	5 (55.6%)	1 (11.1%)	1 (11.1%)	2 (22.2%)	2 (22.2%)
≥7 points	13 (27.7%)	3 (6.4%)	9 (19.1%)	13 (27.7%)	5 (10.6%)
*p*	0.107	0.514	0.49	0.547	0.312
Mechanical ventilation					
Yes (*n* = 20)	10 (50.0%)	1 (5.0%)	2 (10.0%)	6 (30.0%)	3 (15.0%)
No (*n* = 36)	8 (22.2%)	3 (8.3%)	8 (22.2%)	9 (25.0%)	4 (11.1%)
*p*	0.034*	0.549	0.222	0.459	0.487
Neonatal sepsis					
Yes (*n* = 21)	11 (52.4%)	4 (19.0%)	5 (23.8%)	7 (33.3%)	5 (23.8%)
No (*n* = 35)	7 (20.0%)	0	5 (14.3%)	8 (22.9%)	2 (5.7%)
*p*	0.014*	0.016*	0.29	0.29	0.061
RDS					
Yes (*n* = 40)	14 (35.0%)	3 (7.5%)	10 (25.0%)	11 (27.5%)	7 (17.5%)
No (*n* = 16)	4 (25.0%)	1 (6.3%)	0	4 (25.0%)	0
*p*	0.349	0.679	0.024*	0.566	0.08
BPD					
Yes (*n* = 7)	4 (57.1%)	1 (14.3%)	1 (14.3%)	2 (28.6%)	2 (28.6%)
No (*n* = 49)	14 (28.6%)	3 (6.1%)	9 (18.4%)	13 (26.5%)	5 (10.2%)
*p*	0.141	0.423	0.635	0.612	0.163
Intraventricular hemorrhage					
Yes (*n* = 19)	8 (42.1%)	3 (15.8%)	2 (10.5%)	2 (10.5%)	2 (10.5%)
No (*n* = 37)	10 (27.0%)	1 (2.7%)	8 (21.6%)	13 (35.1%)	5 (13.5%)
*p*	0.199	0.108	0.261	0.045*	0.556
Neonatal seizures					
Yes (*n* = 13)	7 (53.8%)	1 (7.7%)	2 (15.4%)	5 (38.5%)	2 (15.4%)
No (*n* = 43)	11 (25.6%)	3 (7.0%)	8 (18.6%)	10 (23.3%)	5 (11.6%)
*p*	0.06	0.664	0.222	0.23	0.519

*statistically significant result.

We found neonatal factors (Apgar score and mechanical ventilation) that were associated both with the developmental status outcomes in the follow-up period and stress hormones in the neonatal period. Apgar score at the 1st and 5th minutes negatively correlated with cortisol level (*r* = −0.41; *p* = 0.007 and *r* = −0.48; *p* = 0.001 respectively). Cortisol was higher in children who had an Apgar score below 7 at the 1st minute (0.619 [0.353; 2.004] μg/dL vs. 0.344 [0.161; 0.738] μg/dL, *p* = 0.057) and at the 5th minute (1.521 [0.637; 2.872] μg/dL vs. 0.371 [0.195; 0.809] μg/dL, *p* = 0.012). In the same time, Apgar score at the 1st and 5th minutes positively correlated with melatonin level (*r* = 0.33; *p* = 0.017 and *r* = 0.36; *p* = 0.009 respectively). Melatonin was lower in children with Apgar scores below 7 at the 1st minute (5.34 [3.60; 7.46] vs. 2.49 [1.75; 6.08] ng/mL, *p* = 0.029) and at the 5th minute (4.73 [2.44; 6.84] vs. 2.48 [1.69; 3.68] ng/mL, *p* = 0.077). The duration of mechanical ventilation positively correlated with cortisol level (*r* = 0.76; *p* = 0.002) and a negatively—with melatonin level (*r* = −0.51; *p* = 0.027) in preterm newborns in the neonatal period.

## Discussion

Currently, the connection between neonatal stress, its physiological mechanisms and its association with clinical, laboratory and instrumental signs of health and development of preterm infants is being actively studied (van Dokkum et al., 2021). This is due to concerns that early exposure to stress may have a negative impact on neurocognitive development (Finegood et al., 2017), and better understanding of these problems can contribute to the improvement of newborn care (van Dokkum et al., 2021).

Although there are strong arguments for the association of neonatal stress with the nervous system development (Walker, 2019; Lavanga et al., 2021; van Dokkum et al., 2021), our research is the first known study that showed the association of laboratory confirmed NICU-related stress with developmental status outcomes in preterm infants at the follow-up period. In addition, we identified the factors associated with developmental status delays in the study group.

Our research showed that delay in the area of communication (32.1%) and problem solving skills (26.8%) were present most often in preterm infants at the corrected age of 24–30 months. They were followed by delayed fine motor (17.9%), personal-social (12.5%), and gross motor skills development (7.1%), which is consistent with numerous studies that indicate a language and cognitive development disorders in prematurely born children (Do et al., 2019; Wolke et al., 2019; You et al., 2019). Delay in one area of development occurred in 26.8% of children of the study group, in two areas of development—in 16.1%, and in 3 or more areas—in 10.7%. Our data are consistent with the results of the Mansson et al. research who found that 20% of preterm infants at the corrected age of 2.5 years had delay in only one area of development (cognition, expressive or impressive language, fine or gross motor skills) according to the Bayley-III scale assessment. Fourteen percent of children had developmental delay in two areas, 13 and 12.5%—in three and five areas, respectively (Mansson and Stjernqvist, 2014).

Our study showed the significant correlations between the ASQ-3 scores of developmental status of preterm infants at the corrected age of 24–30 months and the cortisol and melatonin levels measured during the neonatal period. It indicates that developmental delays in premature children were associated with the experienced stress in the NICU, characterized by elevated stress hormones and reduced anti-stress hormones. Thus, it was proven that ASQ-3 communication and personal-social scores were negatively correlated with the cortisol level (*r* = −0.31; *p* = 0.043 and *r* = −0.35; *p* = 0.022 respectively). At the same time, the total ASQ-3 score, communication, problem solving, and personal-social skills scores were positively correlated with melatonin level (*r* = 0.31, *p* = 0.026; *r* = 0.36, *p* = 0.009; *r* = 0.30, *p* = 0.033, and *r* = 0.32; *p* = 0.022 respectively).

Our study also identified cut-off points for developmental delays in preterm infants at the corrected age of 24–30 months based on the levels of melatonin and cortisol. The ROC-curve analysis confirmed that these values are associated with communication (urinary melatonin level <3.44 ng/mL), problem solving (urinary melatonin level <3.71 ng/mL), and personal-social development delays (salivary cortisol >0.64 mcg/dL). Our results indicate the need for both clinical and laboratory monitoring of stress and pain indicators in the NICU, which are very difficult to distinguish in the neonatal period, and their adequate management can prevent neurodevelopmental delays in preterm children.

Our findings align with Grunau et al. research, which revealed a relationship between a greater number of skin-breaking procedures during the neonatal period and reduced cognitive and motor scores as assessed by the Bayley scale at the age of 8 and 18 months (Grunau et al., 2009). Other authors also revealed by the association of neonatal stress with the emotional, behavioral, and attention deficit disorders in early childhood (18–36 months) (Montirosso et al., 2016; Gaspardo et al., 2018), and at school age (7 to 11 years) (Chau et al., 2019). Valeri et al. showed that early-life stress experienced during critical periods of nervous system development in preterm infants affects brain development by altering its structure, as well as the infant’s behavior, motor skills, and reactivity to stress (Valeri et al., 2015).

Smith et al. revealed that increased exposure to stressors in the NICU was associated with a decrease of brain size in the frontal and parietal regions and altered brain microstructure and functional connectivity in the temporal lobes, which was clinically manifested by the neurobehavioral changes of preterm neonates at the postconceptual age of 40 weeks (Smith et al., 2011). These changes in the structure of the frontal lobe are important for social, concentration and executive development and information processing. Functions related to emotion and attention have been described to originate in the frontal lobe, cerebellum, and cortical areas of the brain that have been reported to be affected by neonatal stress (van Dokkum et al., 2021).

The impact of neonatal stress on the developmental status can be explained through the direct influence of stress hormones, particularly cortisol, on the nervous system formation and maturation in the preterm infants. Premature children in NICU are exposed to the stress, which is accompanied by an increase in cortisol during a critical period of brain development when synaptic connections are being formed and cortical networks are being established (van Dokkum et al., 2021; Pavlyshyn and Sarapuk, 2023). At the same time, positive correlations of the ASQ-3 total scores, communication, problem solving, personal-social development scores of the children with melatonin level confirm its neuroprotective properties (Garofoli et al., 2021; Perrone et al., 2023).

The secondary objectives of our research aimed to identify the factors that were additionally associated with developmental status outcomes in the study group. Our study showed an association between the developmental status of preterm children at the corrected age of 24–30 months and their gestational age. Extremely and very preterm neonates were significantly more likely to have delayed communication and problem-solving skills development compared to moderately preterm children. Other authors also indicate that children born extremely premature have the highest risk of adverse developmental outcomes, with the risk of developmental adversities decreasing with each additional week of gestation through to full-term period (Poulsen et al., 2013; Hanly et al., 2018; Dhamrait et al., 2021). Recent literature data describes that preterm birth is associated with poor outcomes in infants (especially extremely and very preterm, and those who weigh <1,500 g) in various developmental domains, including motor, cognitive, behavioral, and sensory (de Jong et al., 2015; Ionio et al., 2016). There is a dose-dependent relationship between gestational age and poorer school readiness (Dhamrait et al., 2021). Such associations between the gestational age and the developmental status of preterm infants can be explained by the immaturity of the brain in extremely and very premature newborns in combination with the stress experienced in the neonatal period. Preterm birth can affect the process of proliferation, differentiation, migration, and cerebral, cerebellar growth, leading to structural and functional neurodevelopmental disorders (Wallois et al., 2020). At the same time, extremely and very preterm infants are exposed to a greater number of repeated painful treatment and care procedures during a period of rapid brain development and programming and formation of stress systems (D’Agata et al., 2017), and have been shown to experience more pronounced stress in the NICU (van Dokkum et al., 2021).

Our findings also revealed that infants with lower Apgar scores exhibited lower ASQ-3 communication and personal-social development scores at the corrected age of 24–30 months, as evidenced by negative correlations. Additionally, these infants displayed significantly higher levels of cortisol and lower levels of melatonin in the NICU. These data suggest that children with low Apgar scores experience greater stress in the neonatal period, which negatively affects their development. Premature infants with low Apgar scores undergo resuscitation in the delivery room and intensive neonatal care during the first hours of life (D’Agata et al., 2017; Pados, 2019), which can serve as an additional trigger for an excessive long-lasting stress response. It was shown that the rate of mortality, morbidity, and therefore the frequency of neonatal interventions and complications is significantly higher in preterm infants with low Apgar scores (American Academy of Pediatrics, 2015).

Our study revealed an association between the mechanical ventilation in the neonatal period and further development of infants. In particular, children who required mechanical ventilation had significantly more delayed communication, as well as a negative correlation was established between the duration of mechanical ventilation and ASQ-3 gross motor development scores. At the same time, neonatal stress markers were also associated with the duration of mechanical ventilation. The positive correlation of cortisol and negative correlation of melatonin with the duration of mechanical ventilation confirms that this intervention is stressful for preterm infants.

These findings align with those from another study, where the authors reported that invasive ventilation represented a stressful experience for newborns and leads to changes in neuroendocrine system, pain indicators and physiological responses (Bellù et al., 2021). Assisted lung ventilation in newborns is believed to lead to long-lasting persistent pain, which is associated with adverse long-term outcomes (Bellù et al., 2021). This association is confirmed by the results of delayed developmental status of preterm infants in our study. Voss et al. also showed that mechanical ventilation for more than 2 weeks significantly increased the risk of developmental disorders in extremely premature neonates (Voss et al., 2016).

Our study also revealed that longer durations NICU stay and total hospitalization dare associated with the lower ASQ-3 communication scores at the corrected age of 24–30 months, confirming the significant consequences of long-term NICU-related stress for the development and functioning of the preterm infants’ brain (Aita et al., 2017).

Delayed communication and gross motor skills development occurred more often in children who experienced sepsis during the neonatal period compared to those who did not have this condition. Our results are consistent with the data of a meta-analysis by Cai, which indicated that very preterm infants with neonatal sepsis are at the higher risk of cerebral palsy and neurosensory deficits (Cai et al., 2019). Studies show that the developing brain is vulnerable to a systemic inflammatory response including cytokine and free radical activation, as well as to ischemic damage from hypotension and reduced cerebral blood flow. These factors together lead to white matter damage and diffuse lesions of premyelinated oligodendrocytes, which, as was shown to be closely associated with an increased risk of impaired cognitive and motor functions (Hemels et al., 2012; Hentges et al., 2014; Cai et al., 2019).

Maternal age emerged as the factor associated with the developmental status of preterm infants. It was established that an older maternal age was associated with the higher total ASQ-3 scores, as well as improved ASQ-3 communication, and personal-social development scores. The link between maternal age and neonatal outcomes, however, remains controversial in the literature. Tseng et al. reported that maternal age was not associated with major morbidity and long-term neurodevelopmental impairment among very low birth weight infants. The exception was an observed association between older maternal age and delay in language skills, a finding the authors noted requires further investigation (Tseng et al., 2019). In contrast, other recent population-based cohort studies have shown that older maternal age may be beneficial for early child development (Barclay and Myrskylä, 2016; Kato et al., 2017). Today’s older mothers often have a higher socioeconomic status, which is frequently linked with higher education, higher family income, and private health insurance. These factors together create a more optimal environment and contribute to more favorable developmental outcomes for children (Carolan and Frankowska, 2011).

### Practical implications

The health care providers to prevent developmental delays in preterm children should systematically monitor the exposure of preterm infants to stress in the NICU. Minimizing the impact of NICU-related stress on newborns is extremely important. Implementing such developmental care practices as pain management, creation of a healing NICU environment, and family-integrated approach to care can significantly reduce the impact of neonatal stress and therefore improve developmental status in the follow-up period (van Dokkum et al., 2021). Developmental care includes a variety of early intervention strategies that help protect the infant from adverse environmental factors, create a neonatal environment that minimizes stress for the infant, reduces pain, and provides sensory experiences appropriate to the infant’s development (Pierrat et al., 2016). Parental participation in care is one of the most important components of developmental care, which improves the neurological and behavioral development of a child up to 24 months of corrected age (Vanderveen et al., 2009). Neuro-developmental care approaches also help to improve the processes of myelination, neurobehavioral and neurophysiological functioning of the child, promoting cognitive, motor and language development in the follow up period (Soleimani et al., 2020). Montirosso suggest that high quality developmental care in newborns may mitigate behavioral problems in preterm infants at 18 months of age potentially leading to a behavioral profile that is similar to that of full-term peers (Montirosso et al., 2016).

Kangaroo mother care with skin-to-skin contacts is also an important strategy for the care of preterm infants, which minimizes stress and improves the developmental status outcomes. It was found in our previous study that regular and repeated skin-to-skin contacts can lead to a stress-buffering anxiolytic effect for preterm infants in the NICU (Pavlyshyn et al., 2022), while deprivation of pleasant maternal touch in newborns can lead to toxic stress, which is associated with a number of developmental disorders in infants (Bergman, 2019). Skin-to-skin contact helps improve the neurological development of children, their motor, cognitive and behavioral functions, and sleep organization in the follow-up period (Feldman et al., 2014).

## Strengths and limitations

This is the first known study to show the association between the laboratory confirmed NICU-related stress and developmental status disorders in preterm infants during the follow-up period. However, the study has some limitations. Firstly, it was conducted as a single-center descriptive study and was constrained by a small sample size, since not all children who underwent laboratory stress tests in the neonatal period had reached the correct age of 24–30 months at the time of follow-up assessment. With this reason, this study was with single time point for developmental follow-up. Secondly, we did not analyze the cumulative NICU-related stress. There are numerous factors influencing the developmental trajectories of preterm infants, and these factors were considered and analyzed in our study. In addition, there was a lack of control for stress-buffering factors.

## Conclusion

Neonatal stress was associated with long-term developmental disorders in preterm infants at the corrected age of 24–30 months. This association was highlighted by correlations between the neonatal cortisol and melatonin levels with communication, problem-solving skills and personal-social development at the follow up. Levels of melatonin below and cortisol above the certain cut-off values in the neonatal period may serve as one of the predictors of developmental status disorders in preterm neonates. Additionally, factors such as low gestational age, long duration of hospital stay, in particular in the NICU, mechanical ventilation, neonatal sepsis were associated with more frequent developmental delays in the areas of communication, gross motor, and problems solving skills.

## Data availability statement

The raw data supporting the conclusions of this article will be made available by the authors, without undue reservation.

## Ethics statement

The studies involving humans were approved by The Local ethical committee of the I. Horbachevsky Ternopil National Medical University (protocol no 74 issued 28.09.2023). The studies were conducted in accordance with the local legislation and institutional requirements. Written informed consent for participation in this study was provided by the participants’ legal guardians/next of kin.

## Author contributions

HP: Conceptualization, Data curation, Formal analysis, Funding acquisition, Investigation, Methodology, Project administration, Supervision, Validation, Visualization, Writing – original draft, Writing – review & editing. IS: Conceptualization, Data curation, Formal analysis, Investigation, Methodology, Software, Writing – original draft, Writing – review & editing. KK: Writing – original draft, Writing – review & editing, Conceptualization, Formal analysis, Investigation, Methodology, Software.
